# Experimental and Numerical Analyses on the Buckling Characteristics of Woven Flax/Epoxy Laminated Composite Plate under Axial Compression

**DOI:** 10.3390/polym13070995

**Published:** 2021-03-24

**Authors:** Venkatachalam Gopalan, Vimalanand Suthenthiraveerappa, Jefferson Stanley David, Jeyanthi Subramanian, A. Raja Annamalai, Chun-Ping Jen

**Affiliations:** 1 Centre for Innovation and Product Development, Vellore Institute Technology, Chennai 600127, Tamilnadu, India; g.venkatachalam@vit.ac.in; 2 Department of Mechanical Engineering, Karpagam College of Engineering, Coimbatore 614032, Tamilnadu, India; svanand.reg@gmail.com; 3 School of Mechanical Engineering, Vellore Institute Technology, Vellore 632014, Tamilnadu, India; jeffsd2000@gmail.com; 4 School of Mechanical Engineering, Vellore Institute Technology, Chennai 600127, Tamilnadu, India; jeyanthi.subramanian@vit.ac.in; 5 Centre for Innovative Manufacturing Research, Vellore Institute Technology, Vellore 632014, Tamilnadu, India; raja.annamalai@vit.ac.in; 6 Department of Mechanical Engineering and Advanced Institute of Manufacturing for High-Tech Innovations, National Chung Cheng University, Chia-Yi 62102, Taiwan

**Keywords:** green composite, environmental sustainability, buckling, response surface methodology, elastic constants, finite element method

## Abstract

The evolution of a sustainable green composite in various loadbearing structural applications tends to reduce pollution, which in turn enhances environmental sustainability. This work is an attempt to promote a sustainable green composite in buckling loadbearing structural applications. In order to use the green composite in various structural applications, the knowledge on its structural stability is a must. As the structural instability leads to the buckling of the composite structure when it is under an axial compressive load, the work on its buckling characteristics is important. In this work, the buckling characteristics of a woven flax/bio epoxy (WFBE) laminated composite plate are investigated experimentally and numerically when subjected to an axial compressive load. In order to accomplish the optimization study on the buckling characteristics of the composite plate among various structural criterions such as number of layers, the width of the plate and the ply orientation, the optimization tool “response surface methodology” (RSM) is used in this work. The validation of the developed finite element model in Analysis System (ANSYS) version 16 is carried out by comparing the critical buckling loads obtained from the experimental test and numerical simulation for three out of twenty samples. A comparison is then made between the numerical results obtained through ANSYS16 and the results generated using the regression equation. It is concluded that the buckling strength of the composite escalates with the number of layers, the change in width and the ply orientation. It is also noted that the weaving model of the fabric powers the buckling behavior of the composite. This work explores the feasibility of the use of the developed green composite in various buckling loadbearing structural applications. Due to the compromised buckling characteristics of the green composite with the synthetic composite, it has the capability of replacing many synthetic composites, which in turn enhances the sustainability of the environment.

## 1. Introduction

Due to the extensive usage of polymer composites in various structural applications, promoting green composites that are based on natural fibers and bio derived polymers helps to create a sustainable green environment for our next generation. Due to the green materials used in the green composite, it emits no or nil carbon emissions during its destruction than that of a synthetic composite. This, in turn, reduces environmental pollution and provides a sustainable green environment. The evolution of natural fiber composites in loadbearing applications such as the automotive, construction and other sectors encourages the researchers to focus on the natural fiber composites subjected to static and dynamic loads. Due to the structural instability of composite structures, the structure will attain a failure in various ways such as fatigue failure and buckling failure. The possibility of the occurrence of a buckling failure of composite structures due to the axial compression load or thermal load happens often. As the natural fiber polymer composite structures are used in various loadbearing applications, it may be under an axial compression load all along their service. During the entire life cycle of a natural fiber reinforced polymer (NFRP) composite, it must withstand loads and remain stable. However, the structure might be subjected to large compressive loads, which in turn cause a buckling failure. When a failure is due to buckling, an NFRP composite structure fails at a load equal to several times lesser than the material’s yield strength. It is well known that the buckling on a structure depends on the slenderness ratio of the structure. Several research works have addressed the mechanical properties and more physical characteristics of NFRP composites. In addition to the characterization of new materials, it is essential to study their structural performance during the service. Hence it is important to examine the buckling behavior of NFRP composite structures to enhance their usage in various applications.

Leissa [1] presented a brief overview about the buckling characteristics of laminated composite plates and concentrated on various studies such as a plate with interior holes, shear deformation, local effects, nonlinear stress-strain behavior, sandwich construction involving other materials, hygro-thermal effects, external stiffeners, post buckling behavior and the effects of early imperfections. Biggers and Srinivasan [2] addressed the buckling characteristics of a composite plate and identified the improvements achieved during compression/buckling loads of a rectangular composite plate. The authors also discussed the effects of tailoring the laminated composite plates with different boundary conditions, thicknesses, aspect ratios and membrane stiffnesses on the buckling loads.

Kim and Hoa [3] carried out experimental and numerical investigations to study the buckling performance of composite plates subjected to bi-axial loading and proposed the modified rectangular plate specimen. Peining et al. [4] explored the buckling performance of a thin-walled carbon/epoxy laminated circular cylindrical composite shell under combined axial and torsional loading both analytically and experimentally. It was found that the stiffness eccentricity played a major role on the amount of axial buckling load than that of the combined load. Tafreshi [5] presented a buckling and post buckling analysis of a laminated composite cylinder with cut outs when it was subjected to the combined effect of internal pressure and compression and found that the buckling load of a compression loaded cylinder was highly influenced by internal pressure, cut out and orientation.

Zhong and Gu [6] developed an exact solution using the first-order shear deformation theory to investigate the buckling performance of simply supported rectangular plates with a symmetrical cross ply subjected to unidirectional linearly changing in-plane loads. A parametric study was also conducted to probe the buckling load factor due to the effects of the thickness-to-width ratio, the aspect ratio and the modulus ratio.

Priyadarsini and Kalyanaraman [7] addressed the buckling and post buckling characteristics of thin carbon fiber reinforced polymer laminated composite cylindrical shells under load and displacement controlled static and dynamic axial compression both experimentally and numerically. Parametric studies were also carried out by performing a numerical simulation to discover the effect of the different types of loadings, lamina lay-up and amplitudes of imperfection and geometric properties on the ultimate strength of the cylinder under compression. Prabhakaran et al. [8] explored the sound absorption and vibration damping properties of woven flax/epoxy composites and made a comparison with woven glass/epoxy composites. It was found that the sound absorption coefficient and vibration damping capability of flax fiber reinforced composites were higher than that of glass fiber reinforced composites.

Sayer [9] carried out an experimental and numerical investigation to study the ramifications of different ceramic particles such as aluminum oxide (Al_2_O_3_), silicon carbide and boron carbide (B_4_C) on the elastic properties and load carrying capabilities of ceramic particle filled E-glass/epoxy composite plates and found that the critical buckling load of a 10 wt% boron carbide (B_4_C) particle filled composite increased by 42%. Abdellaoui et al. [10] investigated the effects of the number of layers, fiber directions and mechanical properties of a jute fiber reinforced polymer laminated composite both experimentally and numerically. The authors found that the difference between the experimental and calculated results were due to the assumption of a perfect adhesion between the fibers and the matrix. Triki et al. [11] examined the influence of an alkali treatment on the dielectric characteristics of a woven flax fiber reinforced epoxy composite and found that the adhesion of the fibers/matrix highly depended on the cleaning process of the fabric, which affected the dielectric characteristics of the composite. Bensadoun et al. [12] investigated the fatigue behavior of various flax fiber composites (under tension–tension mode) where the flax fiber in the form of textile architectures, a random mat and two laminate configurations were used. It was found that the fiber architecture had a major impact on the fatigue performance of the flax fiber composites where the superior static strength and modulus combinations delivered the best fatigue characteristics.

Rajesh and Pitchaimani [13] investigated the influence of the weaving pattern of plant fiber yarns such as a conventional twisted straight yarn and braided yarn and also the fiber yarn orientation on the mechanical behavior such as tensile, flexural and impact properties. The results revealed that the composite made of the woven fabric with braided jute yarn showed better mechanical properties than that of the composite made with the other fabric having a conventional weaving pattern. It was also found that the composite made of the woven braided fabric exhibited better mechanical properties than that of a random oriented short fiber reinforced composite. Rajesh and Pitchaimani [14] explored the buckling and free vibration behaviors of a natural fiber reinforced composite beam subjected to axial compression experimentally. An experimentally computed critical buckling load was verified with a numerical analysis based on the finite element method. It was found that the buckling strength of the composite laminate was enhanced with the number of layers and it was also observed that the weaving style of a woven fabric affected the critical buckling load in which the basket type weaving model gave a better buckling strength. Suthenthiraveerappa and Gopalan [15] investigated the effect of porosity associated with natural, i.e., plant fiber, composites along with the transversely isotropic characteristics of plant fibers on the elastic constants of both jute and aloe fiber composites. A new methodology was also developed to estimate the elastic constants of uniform and taper polymer laminated composites reinforced with natural fiber.

Rozylo et al. [16] performed a numerical and experimental investigation on the buckling of thin-walled carbon/epoxy laminate composite profiles with top-hat sections under an axial compression and focused mainly on the critical and post critical states. Gopalan et al. [17] carried out experimental and numerical investigations on the dynamic characteristics of uniform plant fiber reinforced polymer laminated composite plates wherein the experimentally determined elastic constants of the composite lamina were used for the numerical simulation based on the hierarchical finite element method. Suthenthiraveerappa et al. [18] addressed the dynamic characteristics of thickness tapered plant fiber reinforced polymer laminated composite plates both numerically and experimentally. A variable size (h) and polynomial degree (p) [h-p] finite element model using the higher-order shear deformation theory was proposed in a numerical simulation. Various parametric studies were also performed using the proposed finite element model by considering the different parameters such as the ply orientation, the aspect ratio and the number of layers. For the numerical simulation, the elastic constants were determined using a new theoretical approach especially for the NFRP laminated composites.

Xu et al. [19] presented a new symplectic analytical approach embedded with the finite element method to investigate the buckling and vibration characteristics of a partially or internally cracked natural fiber reinforced composite plate with corner point supports. The authors stated that a plate with an internal crack would decrease the critical buckling load and the natural frequency more than that of a plate with a surface crack. Chew et al. [20] explored the mechanical performance of a flax epoxy composite, which was made in the form of a helicoidal laminate stacking configuration under out-of-plane and impact loads and found that this kind of stacking configuration absorbed more energy under an impact load than that of cross ply and quasi-isotropic natural fiber reinforced polymer composite laminates. Ebrahimi et al. [21] investigated the buckling behavior of a graphene oxide powder reinforced (GOPR) nanocomposite shell using the mathematical model based on the first-order shear deformation theory and explored that the critical buckling load of GOPR nanocomposite shells increased significantly with the increase of the weight fraction of the graphene oxide powder. Vallala et al. [22] addressed the structural characteristics of natural fiber reinforced composite plates and pressure vessels such as bending, buckling and vibration responses using the developed mathematical model.

Tuni et al. [23] examined in detail which categories of the supply chain were actually associated in a green performance assessment and discussed the various quantitative methods that are suitable for assessing the environmental performance of supply chains. Goh [24] presented a case study and investigated the barriers on adopting low-carbon warehousing in the Asia-Pacific through an elastic net regression analysis. This work also suggested a low-carbon warehousing technique or procedure to control the carbon emissions in warehouses for providing a sustainable green environment. Carbone et al. [25] discussed the development of environmental dynamic capabilities (DCs) in the field of green supply chain management based on the regression analysis performed to enhance the profitability of the companies and to obtain a sustainable supply chain.

Although many research works have discussed the various research aspects of natural fiber composites, the buckling behavior of natural fiber composites have not been explored well either experimentally or numerically. Optimization tools such as the response surface methodology in the determination of the optimum critical buckling load of natural fiber composites are also not often attempted. Very few works have reported the elastic constants of natural fiber composites but the elastic constants of flax fiber composites are not exposed much. To enhance environmental sustainability, the evolution of green composites in all applications is a must. In order to accomplish that evolution, the development of green composites with a high stiffness and strength is essential.

In this work, experimental and numerical analyses on the buckling behavior of woven flax/bio epoxy composites under an axial compression were carried out. The response surface methodology (RSM) was used to frame the various combinations by considering three different factors that were the number of layers, the width of the plate and the ply orientation and in each parameter three levels were followed. Using the RSM approach, twenty samples each having different combinations were framed for the buckling analysis. The critical buckling load and compressive strength of a woven flax/bio epoxy (WFBE) laminated composite was obtained experimentally for three out of the twenty samples, which were framed using the RSM approach. The numerical simulation was then carried out for the same samples using the finite element software ANSYS. The elastic constants needed for the numerical simulation were determined experimentally. The finite element model used in ANSYS was endorsed by comparing the critical buckling loads of the three samples obtained numerically with the experimentally obtained results. The regression equation was then obtained from MINITAB^©^ software. In addition to the comparison of the experimental and numerical results, the results obtained from the regression equation were compared with both the experimental and numerical results. The authors concentrated on the development of a green composite for buckling loadbearing structural applications to enhance environmental sustainability. This work focused on the composite production industries and researchers in the field of composite structures who are keenly looking to shift from synthetic composites to green composites.

## 2. Response Surface Methodology (RSM)

Response surface methodology (RSM) is a powerful statistically-validated predictive model used in this work for the design and analysis of experiments to determine the optimum combination and to find the influence of various factors such as the number of layers, the width of the plate and the ply orientation on the buckling characteristics. A Box–Behnken design (BBD) of the RSM was used for the experimental design in this work. The factors (parameters) and the three levels of each factor considered in the RSM are given in Table 1.

These levels and factors were used in MINITAB software to frame the various combinations and the combinations obtained are shown in the Table 2. These were then further used to carry out the elastic constants and buckling analysis.

Generally, the coded and uncoded units followed in the experimental design were used to define the different factor levels. It is common that coded units are used to do the analysis in MINITAB software and this helps to identify the influence of specific factors on the output response.

## 3. Materials and Fabrication of the Composites

**Figure 1:**
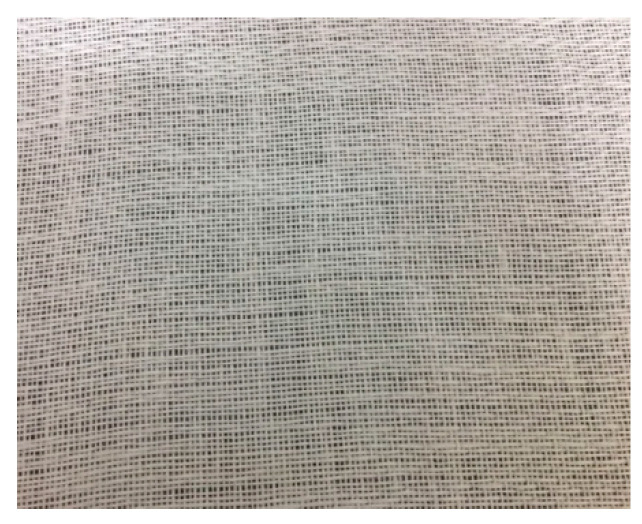
Woven flax fabric.

For this study, bio epoxy and its hardener and plain bidirectional woven flax fiber fabrics were procured from Entropy solutions, Kandel, Germany and Bcomp, CH-1700 Fribourg, Switzerland respectively. The plain woven flax fabric is shown in Figure 1.

**Figure 2:**
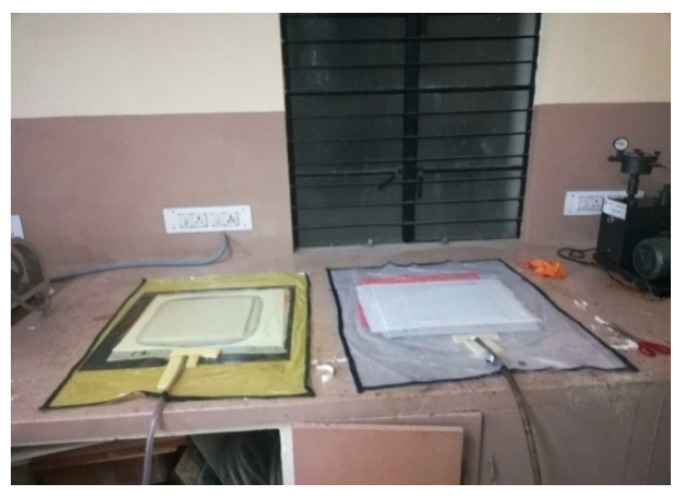
Composite fabrication.

The WFBE laminated composite plate was fabricated using the vacuum assisted hand lay-up technique. Firstly, the woven flax fiber fabrics were kept in an oven for 12 h at 50 °C to eliminate their moisture content. As the flax fibers have a tendency to absorb more resin, extreme care was taken to eliminate the excess resin and to increase the volume fraction of the fiber during the preparation of the WFBE laminated plate. The post curing was then done by keeping the WFBE laminated composite plate in an oven at 80 °C for 3 h. The three WFBE laminated composite plate samples out of twenty samples were fabricated to investigate their buckling characteristics. In order to evaluate the elastic constants of the composite lamina using an impulse excitation of vibration approach, the WFBE laminated composites were also fabricated. The WFBE laminated composite fabrication process using the hand lay-up technique is shown in Figure 2.

## 4. Experimental Investigation

### 4.1. Evaluation of Elastic Constants

**Figure 3:**
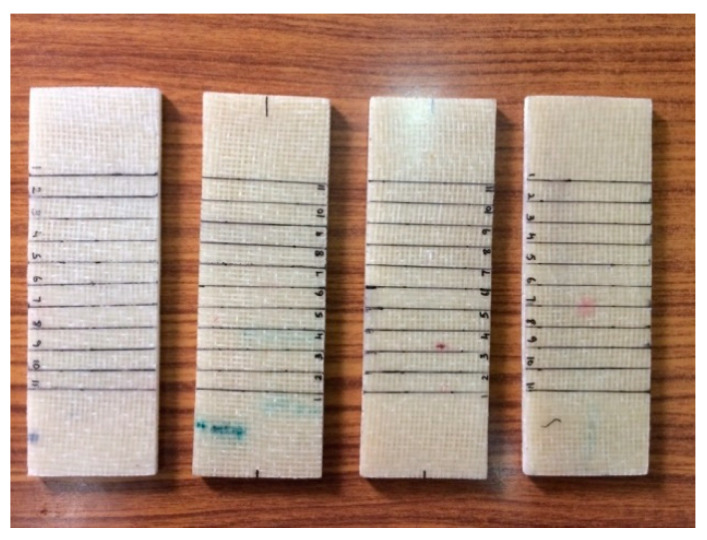
Samples used for the impulsive excitation of vibration.

**Figure 4:**
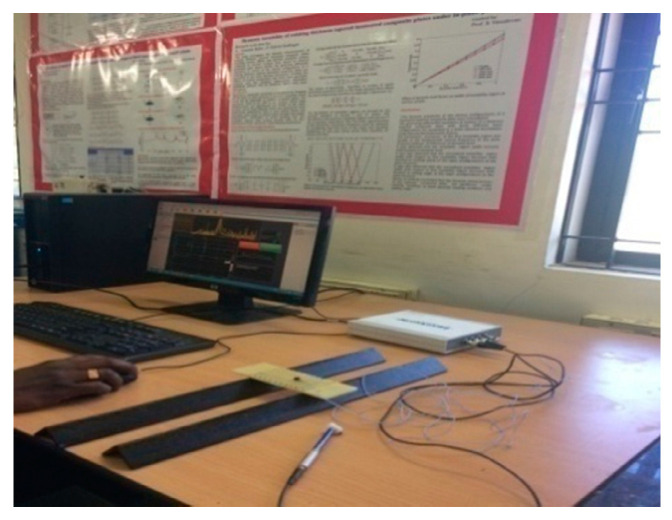
Experimental setup for the fundamental flexure frequency.

**Figure 5:**
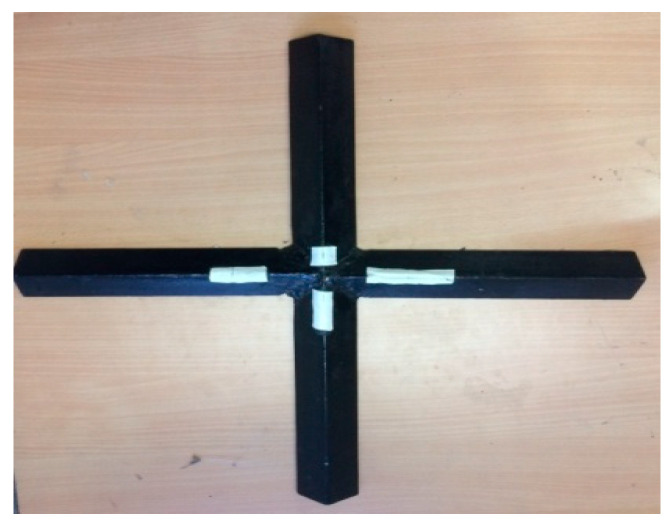
Experimental setup for the fundamental torsional frequency.

To perform the numerical simulation on the buckling analysis of the WFBE laminated composite, the elastic constants of the WFBE composite lamina was needed. The elastic constants needed for the numerical analysis were the in-plane shear modulus, the longitudinal and transverse elastic modulus and Poisson’s ratios. In this work, a nondestructive testing method (namely, the impulsive excitation of vibration approach based on ASTM E1896) was used for the evaluation of the elastic constants of the WFBE composite lamina. The dimensions of the specimens were taken from ASTM E1876 and the fabricated WFBE laminated composite specimens for testing are shown in the Figure 3. The experimental setups used for the impulsive excitation of vibration to determine the flexural and torsional resonant frequencies of the specimen are shown in Figure 4 and Figure 5.

### 4.2. Experimental Buckling Analysis

**Figure 6:**
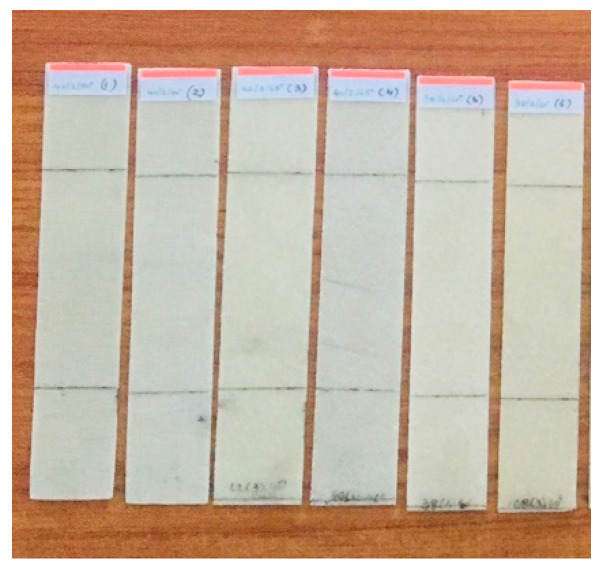
Samples of the woven flax/bio epoxy (WFBE) laminated composite.

The critical buckling load and compression strength of the WFBE laminated composites were determined experimentally by carrying out the buckling test under an axial compression using an Instron 8801 (Instron, Norwood, MA, USA) model machine. The experimental buckling test was conducted on three out of twenty samples, as framed in Table 2. The buckling test samples were cut from the fabricated composite plates. The buckling test samples had 200 mm in length in common. The width, number of layers and orientation were taken, as based on Table 2. The change in the number of layers changed the thickness of the samples. Table 3 shows the thicknesses of the samples that were formulated by taking the mean thickness. The fabricated WFBE laminated composite samples are shown in Figure 6.

**Figure 7:**
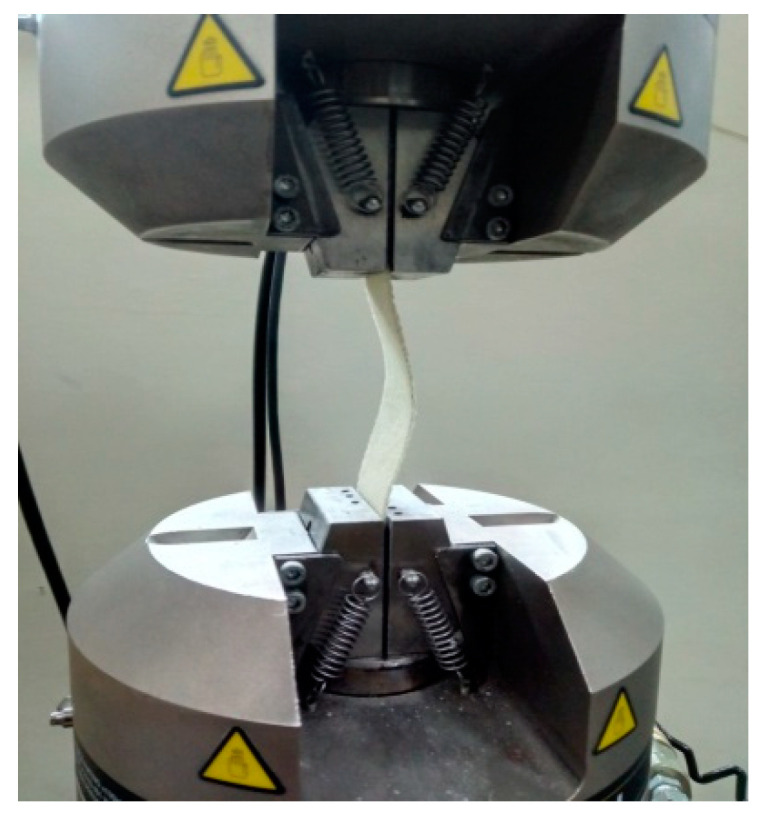
Buckling of a composite sample.

The samples were clamped at 50 mm on both sides of its length by wedge grips and the remaining length of 100 mm was the effective buckling length. Figure 7 shows the buckling test carried out on a composite sample.

## 5. Results and Discussions

### 5.1. Elastic Constants

In order to carry out the numerical simulation on the buckling of the WFBE composite, the elastic constants and the material properties of the WFBE composite needed to be determined. The elastic constants were determined using the impulse excitation of vibration approach. The elastic constant evaluation was done based on ASTM E1876.

The fundamental flexural (*f_f_*) and torsional frequencies (*f_t_*) were obtained using this approach, which are given in Table 4. The fundamental flexural (*f_f_*) and torsional frequencies (*f_t_*) were used to determine the elastic constants of the WFBE composite lamina such as the dynamic Young’s modulus and shear modulus, respectively, by using the formulae given in ASTM E1896. It was then further used to calculate the Poisson ratio. Table 5 shows the determined elastic constants of the WFBE composite lamina needed for the numerical simulation on the buckling of the WFBE composite.

### 5.2. Experimental Buckling

The buckling under axial compression was carried out experimentally for the three samples (2, 4 and 20) from Table 2. The critical buckling load of the composites is discovered from the load versus the displacement curves. The critical buckling load was identified as the first point of the load versus the deflection curve departed straight line. The critical buckling load and the compressive strength for the three samples are given in Table 6.

### 5.3. Numerical Buckling Analysis

**Figure 8:**
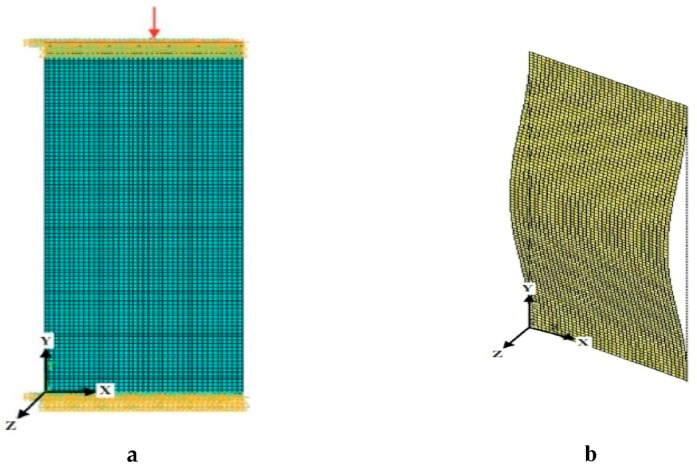
Meshed model (**a**); buckled model under a clamped–clamped end condition (**b**).

The numerical analysis was carried out to investigate the buckling performance of WFBE laminated composite samples under axial compressive loads. The three samples (2, 4 and 20) that were used for the experimental buckling test were also considered for the numerical analysis. On performing the numerical simulation using ANSYS16, the critical buckling load and the load factor were determined for the three composite samples. In the numerical simulation, an Eigen value buckling analysis was performed for the three composite samples. The element used in the modeling of the laminated composite in this analysis was SHELL181, which is a four-node element with six degrees of freedom (DOF) at each node. Under sections, the lay-up method was used to specify the number of layers and the orientation of the specimen. The compression load was applied uniformly as a pressure of 125 bar (50 kN). Figure 8a shows a meshed model subjected to an axial compression load under a clamped–clamped end condition. Figure 8b shows a buckled model under a clamped–clamped end condition.

The critical buckling load was discovered by multiplying the load factor with the amount of pressure applied. The comparison table of critical buckling loads obtained experimentally and numerically using ANSYS16 is given in Table 7.

### 5.4. Comparison of Experimental and Numerical Results

The finite element model was endorsed by comparing the critical buckling loads obtained experimentally and numerically using ANSYS16 for the considered same three samples.

As the validation showed a positive sign, the numerical simulation was performed for all of the 20 samples from Table 2. Table 8 shows the critical buckling loads obtained numerically using ANSYS16 for all of the 20 samples. The results obtained from the numerical simulation (ANSYS16) were used to perform the ANOVA assessment in MINITAB software. From the numerical simulation results, it was found that the maximum and minimum critical buckling loads were obtained for the samples in which the width was 42 mm and the number of layers were four and two, respectively.

In general, the inclusion of layers to composites enhances the load carrying ability of composites. Accordingly, composites with four layers had the best ability to defy the buckling load. In general, composite specimens with a 42 mm width showed a higher resistance to the buckling load. The change in the orientation of the composite showed a significant change in the critical buckling load. The specimens with the 60° and 90° ply orientations showed a greater critical load whereas the 45° ply orientations showed lower buckling loads compared with the 60° and 90° ply orientations of which the 90° ply orientations showed the highest buckling loads. Therefore, the change in the varying parameters (layers, width and orientation) of composites creates a constructive cause on the buckling loadbearing ability of the composites.

### 5.5. Analysis of Variance (ANOVA) Assessment for the Regression Model and Regression Equation

**Figure 9:**
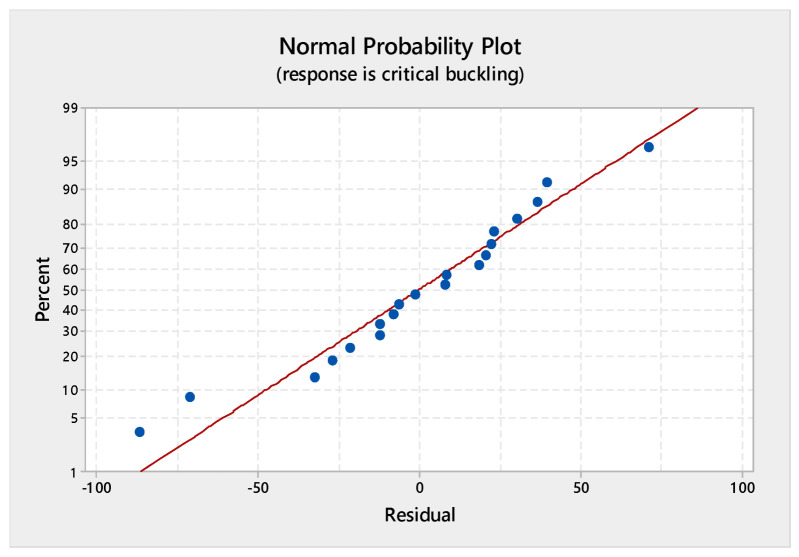
Normal probability plot.

**Figure 10:**
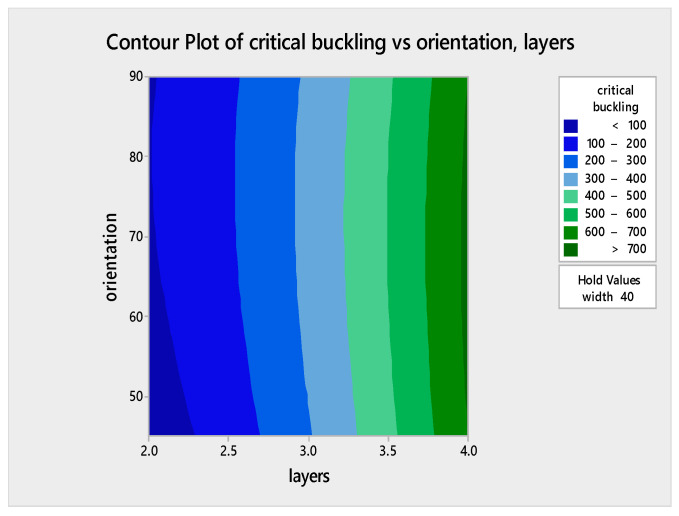
Contour plot of critical buckling versus orientation, layers.

**Figure 11:**
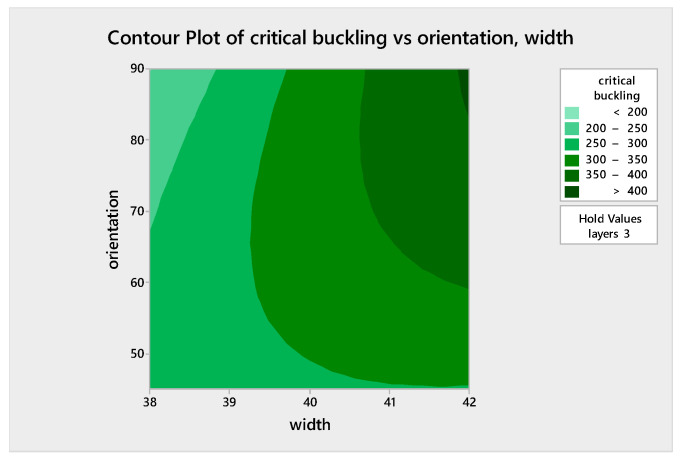
Contour plot of critical buckling versus orientation, width.

**Figure 12:**
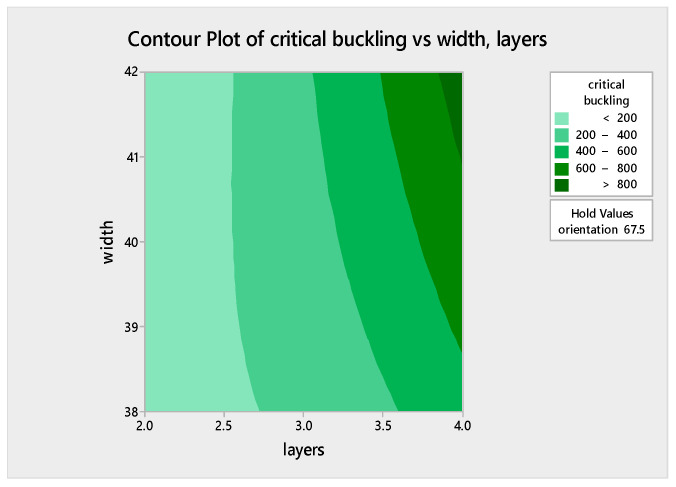
Contour plot of critical buckling versus width, layers.

An ANOVA assessment was carried out via MINITAB software to probe the effect of the three factors on the critical buckling of the WFBE composites. From this evaluation, a normal probability plot and contour plots for varying parameters such as the contour plot of the critical buckling versus orientation (layers), the critical buckling versus orientation (width) and the critical buckling versus width (layers) were obtained. The normal probability plot for the response of the critical buckling load is given in Figure 9. The contour plots shown in Figure 10, Figure 11 and Figure 12 exemplify the influence of various factors such as the number of layers, the width of the plate and the ply orientation on the critical buckling of the WFBE composites. These contour plots demonstrated the interaction between any two of the three parameters on the critical buckling of the WFBE composites.

From the normal probability plot for the response of the critical buckling load given in Figure 9, it was noted that all of the data points fell closely along the straight distribution line. This clearly represented that the plot obtained had a good fit with the data.

Figure 10 clearly depicts that when the number of layers increased, the critical buckling value also increased for the width of 40 mm. It was noted that the influence of orientation was less when compared with the influence of the number of layers. On close monitoring, it was observed that when the orientation was above 60° and the number of layers was above four, a higher critical buckling load was obtained. Figure 11 shows a high critical buckling load when the width and orientation were 42 mm and 90°, respectively, under the constant value of three layers. On the contour plot shown in Figure 12 under the ply orientation of 67.5°, it was found that the maximum critical buckling load was obtained when the number of layers was close to four and the width was above 41 mm. It was also clear that the influence of the number of layers was higher than that of the width on the critical buckling load. That is, when the number of layer increased, the critical buckling load of the WFBE composites increased.

As the comparison of the results of the experimental and numerical (FEA) buckling analyses showed a good correlation, a simulation was performed using ANSYS16 and results were obtained for all of the 20 combinations. These results were further used to create a regression equation using MINITAB17 software. The regression equations (RE) given in Equation (1) were obtained for the evaluation of the critical buckling of the WFBE composites through the ANOVA technique using MINITAB software. The independent variables of Equation (1) were the parameters considered in this work such as the number of layers, the width of the plate and the ply orientation. The coefficient values associated with each independent variable in Equation (1) represented the nature of the influence of various parameters on the critical buckling of the WFBE composites. Using this regression equation, the critical buckling load of the WFBE composites was obtained by the substitution of the corresponding value of all of the three parameters considered in this work. This helped to determine the intensity of the influence of the three parameters on the critical buckling load of the WFBE composites.

Finally, the critical buckling load for all of the combinations was calculated using the obtained regression equation. It was then compared with the results obtained using FEA software and this comparison is presented in Table 9.

The regression equation in uncoded units is given as:

(1) $$\begin{array}{cc} \mathrm{Critical}\;\mathrm{Buckling}\;\mathrm{Load}\;= & \;14868\;+\;632\;\mathrm{layers}\;-\;851\;\mathrm{width}\;+\;33.3\;\mathrm{orientation}\;+\;9.5\;\mathrm{layers}\;\times \;\mathrm{layers}\\ + & \;12.14\;\mathrm{width}\;\times \;\mathrm{width}\;+\;0.0034\;\mathrm{orientation}\;\times \;\mathrm{orientation}\;-17.6\;\mathrm{layers}\;\times \;\mathrm{width}\\ + & \;2.858\;\mathrm{layers}\;\times \;\mathrm{orientation}\;-\;1.004\;\mathrm{width}\;\times \;\mathrm{orientation}\; \end{array}$$

From Table 9, by the comparison of the results, it was evident that the percentage of deviation was between 3–5% for most of the cases and, except for a few, the percentage of deviation was less than 10%. Hence it established the credibility of the calculated regression equation and the developed regression model. This in turn showed the finite element model was stable and the same model was used for a similar kind of analysis. This regression equation further helped to determine the critical buckling load of all possible combinations other than the 20 combinations from Table 2.

Apart from the benefit of loadbearing capabilities, this green composite reduces pollution and is supportive in the development of a green environment. As the composite is biodegradable in nature, it is suggested for loadbearing applications such as civil construction, automobiles, home appliances and rail, aircraft and marine applications by replacing the synthetic composites.

## 6. Conclusions

This work attempted a development of a sustainable green environment using a green composite for various buckling loadbearing structural applications. In this study, numerical and experimental investigations were performed on the buckling characteristics of a (WFBE) laminated composite by considering three different parameters (i.e., layers, width and orientation). The RSM approach was adopted in this work to design and analyze the buckling characteristics of the WFBE composite and also to establish the effect of various parameters on the buckling characteristics of the WFBE composite. The numerical model was endorsed by comparing the experimental and numerical results of the WFBE composite. It was found that the critical buckling loads obtained from the numerical solutions and the experimental data were in good agreement. The regression equation was framed using the numerical results obtained from the validated numerical model (FEA). Finally, a comparison was made between the numerical results obtained through the FEA and the results obtained using the regression equation.

It was found that the obtained regression equation was used to compute the approximate critical buckling load of all of the combinations of the flax bio epoxy composite laminates without undergoing any experimental and numerical analyses. It was understood that the load carrying ability and elasticity moduli of the composites were notably influenced by the change in the number of layers, width and orientation of the composites. With appropriate consideration to composite design and structural geometry, natural fiber composites may establish a viable substitute to traditional structural materials. These initial results of the structural properties and design schemes demonstrate promise for light commercial and residential building applications; however, more research is necessary.

## Figures and Tables

**Table 1:** Levels and factors.

FACTORS	LEVELS		
	−1	0	1
LAYERS (X1)	2	3	4
WIDTH (X2)	38	40	42
ORIENTATION (X3)	45	90	60

**Table 2:** Response surface methodology.

RUN ORDER	LAYERS (X1)		WIDTH (X2)		ORIENTATION (X3)	
	CODED	UNCODED	CODED	UNCODED	CODED	UNCODED
1	−1	2	0	40	0	90
2	−1	2	0	40	0	90
3	0	3	1	42	−1	45
4	−1	2	0	40	−1	45
5	1	4	−1	38	1	60
6	0	3	−1	38	1	60
7	1	4	0	40	0	90
8	0	3	1	42	1	60
9	1	4	0	40	0	90
10	0	3	−1	38	−1	45
11	−1	2	−1	38	0	90
12	1	4	1	42	1	60
13	−1	2	0	40	0	90
14	−1	2	1	42	0	90
15	1	4	−1	38	−1	45
16	1	4	1	42	−1	45
17	−1	2	0	40	0	90
18	−1	2	0	40	0	90
19	0	3	0	40	0	90
20	−1	2	0	40	1	60

**Table 3:** Mean thickness of the specimens.

S.NO	NO OF LAYERS	THICKNESS
1	2	1.5
2	3	2.1
3	4	2.9

**Table 4:** Flexural (*f_f_*) and torsional frequencies (*f_t_*).

FLEXURAL FREQUENCIES (*f_f_*)		TORSIONAL FREQUENCIES (*f_t_*)	
WARP	WEFT	WARP	WEFT
637.750	650.000	756.250	731.250

**Table 5:** Elastic constants of the WFBE composite lamina.

ELASTIC CONSTANTS	EXPERIMENTAL RESULTS
E_1_ (GPa)	5.968
E_2_ (GPa)	5.392
G_12_ (GPa)	1.53
µ_12_	0.396

**Table 6:** Experimental critical buckling results.

S.NO	SAMPLE O	SPECIMEN LABEL	MAXIMUM CRITICAL BUCKLING LOAD (N)	COMPRESSIVE STRENGTH (MPa)
1.	2	40/2/90	80.32322	1.33872
2.	20	40/2/60	88.22679	1.47045
3.	4	40/2/45	67.88969	1.13149

**Table 7:** Comparison table of critical buckling loads obtained experimentally and numerically using ANSYS16.

S.NO	SAMPLE NO	SPECIMEN LABEL	CRITICAL BUCKLING LOAD (N)		% OF DEVIATION
			EXPERIMENTAL	NUMERICAL (ANSYS16)	
1.	2	40/2/90	80.32	85.88	6.92
2.	20	40/2/60	88.23	85.98	2.55
3.	4	40/2/45	67.89	68.71	1.21

**Table 8:** Numerical buckling analysis results using ANSYS16.

S.NO	SPECIMEN LABEL	LOAD FACTOR	CRITICAL BUCKLING LOAD, N
1	40/2/90	0.6878	85.875
2	40/2/90	0.6878	85.875
3	42/3/45	2.47294	309.1175
4	40/2/45	0.54964	68.705
5	38/4/60	3.2598	407.475
6	38/3/60	2.1580	269.75
7	40/4/90	4.4531	556.6375
8	42/3/60	2.1187	264.83
9	40/4/90	4.4531	556.6375
10	38/3/45	1.2908	162.26
11	38/2/90	0.8356	104.4552
12	42/4/60	7.8697	983.722
13	40/2/90	0.6878	85.875
14	42/2/90	0.7944	99.305
15	38/4/45	3.0548	381.856
16	42/4/45	7.6142	951.77
17	40/2/90	0.6878	85.875
18	40/2/90	0.6878	85.875
19	40/3/90	1.972	248.41
20	40/2/60	0.6878	85.9786

**Table 9:** Comparison of the critical buckling load of the finite element analysis model (FEA) and the regression equation.

S.NO	SPECIMEN LABEL	CRITICAL BUCKLING, (N)		% OF DEVIATION
		FEA	REGRESSION EQUATION	
1	40/2/90	85.88	89.87	4.45
2	40/2/90	85.88	89.87	4.45
3	42/3/45	309.12	285.29	8.35
4	40/2/45	68.71	89.80	4.35
5	38/4/60	407.48	459.04	7.23
6	38/3/60	269.75	258.76	6.58
7	40/4/90	556.64	540.49	2.99
8	42/3/60	264.83	285.86	7.36
9	40/4/90	556.64	540.49	2.97
10	38/3/45	162.26	197.93	8.02
11	38/2/90	104.46	117.87	11.38
12	42/4/60	983.72	415.70	12.45
13	40/2/90	85.88	89.87	4.45
14	42/2/90	99.31	94.89	4.65
15	38/4/45	381.86	355.34	7.46
16	42/4/45	951.77	372.27	14.55
17	40/2/90	85.88	89.87	4.45
18	40/2/90	85.88	89.87	4.45
19	40/3/90	248.41	289.70	12.26
20	40/2/60	85.98	77.63	10.75

## Data Availability

The data presented in this study are available on request from the corresponding author.
